# Persistent *Aspergillus fumigatus* infection in cystic fibrosis: impact on lung function and role of treatment of asymptomatic colonization—a registry-based case–control study

**DOI:** 10.1186/s12890-022-02054-3

**Published:** 2022-07-05

**Authors:** Axel Blomquist, Malin Inghammar, Mahasin Al Shakirchi, Petrea Ericson, Christina Krantz, Marcus Svedberg, Anders Lindblad, Lisa I. Påhlman

**Affiliations:** 1 Department of Clinical Sciences Lund, Section for Infection Medicine, Skåne University Hospital, Lund University, BMC B14, 221 84 Lund, Sweden; 2 Stockholm Cystic Fibrosis Centre, Karolinska University Hospital Huddinge, Department of Clinical Science, Intervention and Technology, Division of Paediatrics, Karolinska Institute, Stockholm, Sweden; 3 Department of Respiratory Medicine, Sahlgrenska University Hospital, 413 45 Gothenburg, Sweden; 4 Department of Women’s and Children’s Health, Uppsala University, Uppsala, Sweden; 5 Department of Paediatrics, Institute of Clinical Science at The Sahlgrenska Academy, University of Gothenburg, Gothenburg, Sweden; 6 Wallenberg Centre for Molecular Medicine, Lund University, Lund, Sweden

**Keywords:** Cystic fibrosis, *Aspergillus fumigatus*, Lung function, Antifungal treatment

## Abstract

**Background:**

*Aspergillus fumigatus* is the most common filamentous fungus isolated from the airways of people with cystic fibrosis (CF). The aim of this study was to investigate how chronic *A. fumigatus* colonization affects lung function in people with CF, to identify risk factors for colonization, and to evaluate antifungal treatment of asymptomatic *Aspergillus* colonization.

**Methods:**

Data from 2014–2018 was collected from the Swedish CF registry and medical records. Baseline data before the start of *A. fumigatus* colonization was compared with the two succeeding years to evaluate how colonization and treatment affected lung function and other clinical aspects.

**Results:**

A total of 437 patients were included, of which 64 (14.6%) became colonized with *A. fumigatus* during the study period. Inhaled antibiotics was associated with *A. fumigatus* colonization (adjusted OR 3.1, 95% CI 1.6–5.9, *p* < 0.05). Fungal colonization was not associated with a more rapid lung function decline or increased use of IV-antibiotics compared to the non-colonized group, but patients with *A. fumigatus* had more hospital days, a higher increase of total IgE, and higher eosinophil counts. In the *Aspergillus* group, 42 patients were considered to be asymptomatic. Of these, 19 patients received antifungal treatment. Over the follow up period, the treated group had a more pronounced decrease in percent predicted Forced Expiratory Volume in one second (ppFEV1) compared to untreated patients (− 8.7 vs − 1.4 percentage points, *p* < 0.05).

**Conclusion:**

Inhaled antibiotics was associated with *A. fumigatus* colonization, but no association was found between persistent *A. fumigatus* and subsequent lung function decline. No obvious benefits of treating asymptomatic *A. fumigatus* colonization were demonstrated.

## Background

Cystic fibrosis (CF) is one of the most common life-shortening monogenetic diseases. It is caused by a mutation in the cystic fibrosis transmembrane conductance regulator (*CFTR*) gene, resulting in a deficient or dysfunctional chloride channel [1]. The dominant clinical symptoms in people with CF originate from the airways, where thick mucus, impaired ciliary function, and altered airway defence provide a breeding ground for microbes, leading to infection, inflammation and progressive lung function decline [2]. People with CF are often chronically infected with microbes such as *Pseudomonas aeruginosa* and *Staphylococcus aureus* [1]. In addition to bacteria, fungi such as *Candida* and *Aspergillus* species are frequently recovered from CF airways [3]. The prevalence of *A. fumigatus* among people with CF varies widely across studies [4], but overall, about one third of the patients tested positive for *A. fumigatus* in at least one airway culture over 12 months, and about one tenth were defined as chronically colonized in previous studies [5–9]. In people with CF, *A. fumigatus* causes infections ranging from asymptomatic colonization to *Aspergillus* bronchitis, as well as the hypersensitivity reaction known as Allergic Bronchopulmonary Aspergillosis (ABPA) [10]. While ABPA is associated with a more rapid decline in lung function [11], the importance of *A. fumigatus* colonization, and in particular asymptomatic carriage and the role of antifungal treatment, remains unclear. Some studies have shown that persistent *A. fumigatus* colonization is associated with more frequent exacerbations [3, 12, 13], lung function decline [3, 5, 12, 13], and lower respiratory-related quality of life questionnaires scores [14]. In contrast, other studies have not found a relationship between *Aspergillus* colonization and a lower lung function [15–17].

Regarding treatment of *A. fumigatus* colonization, the role of antifungal therapy is largely unknown. To our knowledge, there are only two previous studies that have evaluated whether or not antifungal treatment is beneficial, showing diverging results [18, 19]. At present, there is no consensus on whether *Aspergillus* colonization in CF should be treated or not.

In Sweden, the care of people with CF is centralized to four regional CF centres. Regarding asymptomatic *A. fumigatus* colonization, the treatment recommendations differ between the CF centres. Two centres (Stockholm and Lund) treat these patients with antifungals, most commonly with Posaconazole, while the two other centres (Gothenburg and Uppsala) do not treat asymptomatic *A. fumigatus* colonization.

The aim of this study was to identify risk factors for *A. fumigatus* colonization, to analyse how colonization affects lung function, and to evaluate antifungal treatment of asymptomatic *A. fumigatus* colonization.

## Methods

### Study design and participants

In this retrospective study, the study population consisted of patients registered in the Swedish CF registry between the years 2014–2018. The Swedish CF registry is a national quality registry for children and adults with CF. It holds information on demography, clinical and health parameters, and therapy. All four CF centres in Sweden report to the registry, covering > 90% of all patients in Sweden. This study includes patients registered from 2014, when information about antifungal treatment was included in the registry, until 2018, when the CFTR modulator ivacaftor/lumacaftor was introduced in Sweden. Patients under the age of six were excluded due to missing or unreliable spirometry measurements. Patients with ongoing *A. fumigatus* colonization at the start of the study period were excluded since no *A. fumigatus*-free baseline could be identified. Patients who had undergone organ transplantation, deceased patients, patients lacking relevant annual assessments, and patients with ABPA at baseline or an uncertain *A. fumigatus* colonization status were also excluded (Fig. 2).

Data regarding demographics, CFTR genotype, comorbidities, airway pathogens, treatment, lung function, hospitalization days and lab values were retrieved from the Swedish CF registry, whereas data about *A. fumigatus* colonization status, airway symptoms related to *A. fumigatus* colonization and information about antifungal treatment were taken from the patients’ medical records.

**Fig. 1:**
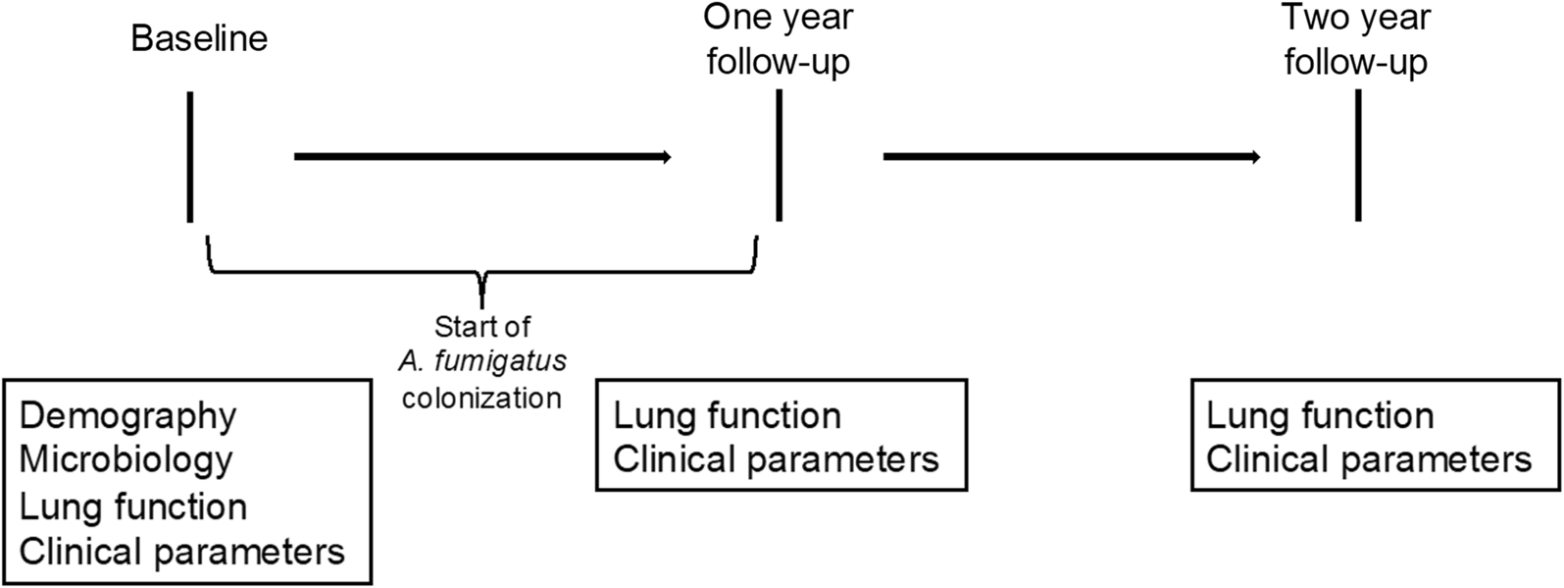
Study design. Baseline data were collected from the annual assessment preceding the start of *A. fumigatus* colonization, and compared to data from the two following annual assessments after approximately 12 and 24 months, respectively. For patients that became colonized with *A. fumigatus*, colonization started at some point between baseline and the one-year follow-up

To identify risk factors for *A. fumigatus* colonization, demographic and clinical baseline data (Table 1) were collected from the last annual assessment preceding the *A. fumigatus* colonization. Hence, the actual colonization started sometime within the next 12 months following baseline. To evaluate the impact of *A. fumigatus* colonization and the role of antifungal treatment, baseline data regarding the patients’ lung function and other clinical aspects were compared with the two succeeding annual assessments after approximately 12 and 24 months, respectively (Fig. 1). Patients without persistent *A. fumigatus* colonization constituted a non-colonized control group. Baseline data for the non-colonized group were collected from 2015. If data were missing, the annual assessments from 2014 or 2016 were used as baseline.

**Table 1:** Baseline characteristics

Baseline characteristics	*Aspergillus fumigatus*	No persistent *Aspergillus fumigatus*	*p*-value
	n (%)	n (%)	
Total	64 (15)	373 (85)	
*Demographics and CF characteristics*			
Age, years (mean ± SD)	21.5 ± 12.8	23.9 ± 13.4	0.18
Female sex	30 (47)	178 (48)	0.90
Homozygous DeltaF508	35 (55)	161 (43)	0.09
CF-related diabetes	15 (23)	62 (17)	0.19
Pancreatic insufficiency	61 (95)	320 (86)	**0.035**
*Microorganisms*			
Chronic PsA colonization	17 (28)	155 (42)	**0.040**
*Treatments*			
Inhaled antibiotics	33 (54)	118 (33)	**0.001**
Inhaled corticosteroids	15 (23)	122 (33)	0.12
Macrolides	12 (19)	136 (37)	**0.005**
Inhaled rhDNase	22 (34)	107 (29)	0.38
*Lung function and other clinical parameters*			
ppFEV1 (mean ± SD)	81.2 ± 21.9	79.1 ± 22.7	0.50
ppFVC (mean ± SD)	94.7 ± 16.1	93.5 ± 17.1	0.59
Number of IV-antibiotics (median (IQR))	1 (0–2)	1 (0–3)	0.66
Hospitalization days (median (IQR))	0 (0–2)	0 (0–0)	0.081
Total IgE (median (IQR))^a^	40.0 (10.5–77.0)	41.5 (16.5–120.0)	0.39
Eosinophils (median (IQR))^b^	0.3 (0.1–0.4)	0.2 (0.1–0.3)	0.086

Patients who developed colonization with *A. fumigatus* compared to those who remained aspergillus-free

*IQR* Interquartile Range; *PsA Pseudomonas aeruginosa*; *ppFEV1* percent predicted Forced Expiratory Volume in one second; *ppFVC* percent predicted Forced Vital Capacity

Bold text indicating *p*-value < 0.05

^a^n = 273

^b^n = 294

Lung function was measured by the spirometry values percent predicted Forced Expiratory Volume in one second (ppFEV1) and percent predicted Forced Vital Capacity (ppFVC) according to the global lung function initiative (GLI) [20]. The change in lung function was calculated as the difference in ppFEV1 (∆ppFEV1) and ppFVC (∆ppFVC) between baseline and follow-up and expressed as percentage points.

### Definitions

Persistent *A. fumigatus* colonization was defined as growth of *A. fumigatus* in two or more airway cultures during a period of 12 months [3, 8, 12]. Patients with a single airway culture positive for *A. fumigatus* were included in the non-colonized group. The definition of chronic *P. aeruginosa* infection in the Swedish CF registry was based on the Leeds criteria from 2003, i.e. growth of *P. aeruginosa* in ≥ 50% of airway cultures during the last 12 months [21]. Symptomatic *A. fumigatus* colonization was defined as airway symptoms that were assessed to be caused by *A. fumigatus* according to the attending physician.

### Statistical analysis

Independent-Samples T Test or Paired-Samples T Test was used for the quantitative parametric variables, and Mann Whitney U Test for quantitative non-parametric variables. Qualitative variables were analysed by cross tabulation analysis. Multivariate logistic regression was used to assess risk factors for *A. fumigatus* colonization. Pancreatic insufficiency, treatment with inhaled antibiotics and macrolide treatment were included in the analysis based on observed differences between the *Aspergillus* and non-colonized groups in this study, whereas age, chronic *P. aeruginosa* colonization and treatment with inhaled corticosteroids were included based on previous knowledge about risk factors for *A. fumigatus* colonization [3, 8, 9, 14, 15, 22]. In addition, ppFEV1 was included in the analysis to adjust for lung function at baseline. Collinearity was assessed by variance inflation factor (VIF). Multivariate linear regression was used to estimate the association between lung function decline and *A. fumigatus* colonization, adjusted for antifungal treatment as well as potential confounders previously known to affect lung function decline; gender, pancreatic insufficiency and chronic *P. aeruginosa* colonization [23, 24]. The limit for statistical significance was set to 5% (*p* = 0.05). Analyses were performed using SPSS, version 25 (SPSS, Armonk, NY, USA).

## Results

### Study population and baseline characteristics

**Fig. 2:**
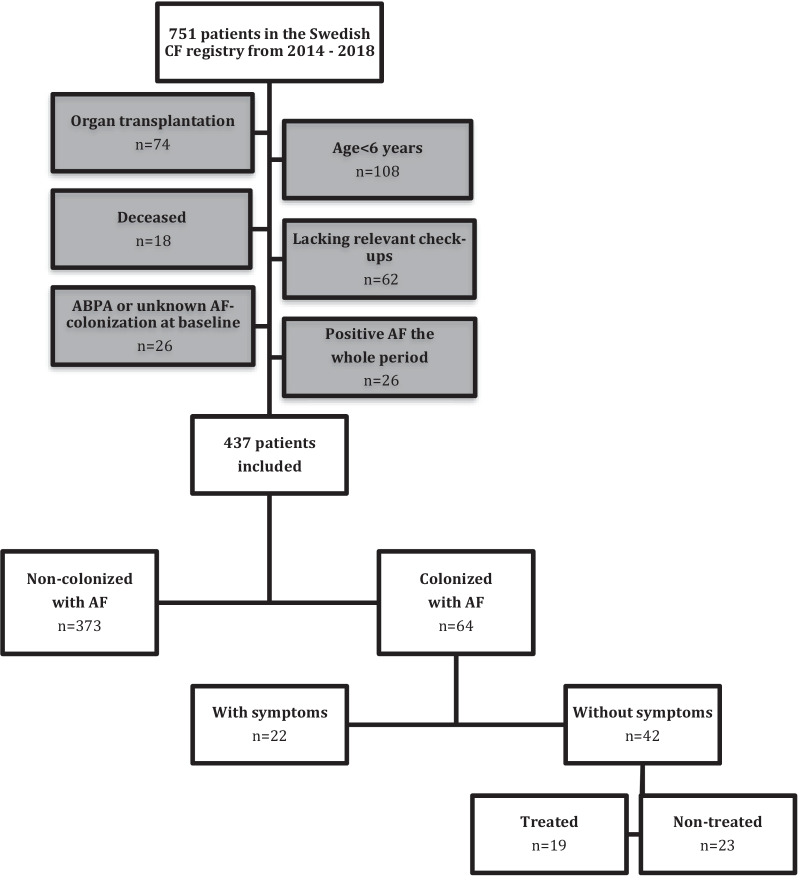
Flowchart of included and excluded patients in the study. *ABPA* Allergic Bronchopulmonary Aspergillosis. *AF Aspergillus fumigatus. CF* Cystic fibrosis

The Swedish CF registry had 751 patients registered during the study period. Of these, 314 patients were excluded (Fig. 2). Of the 437 included patients, 64 (14.6%) met the criteria of becoming chronically colonized by *A. fumigatus* at some point during the study period. The remaining non-colonized group of 373 patients included 37 patients (9.9%) that were positive for *Aspergillus* in the CF registry, but that didn’t fulfil the criteria of persistent colonization according to data in the medical journal.

**Fig. 3:**
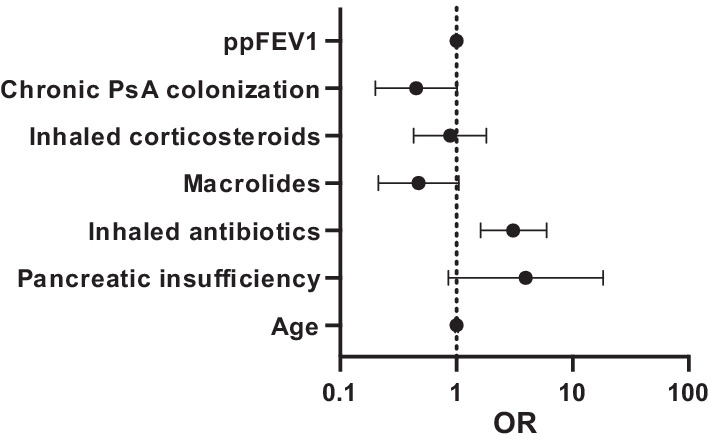
Association between baseline characteristics and chronic *A. fumigatus* colonization estimated by multivariate logistic regression. The forest plot shows odds ratio (OR) and 95% confidence intervals. *N* = 407. *PsA Pseudomonas aeruginosa*; *ppFEV1* percent predicted Forced Expiratory Volume in one second

At baseline, the *Aspergillus* and non-colonized groups were comparable with regards to demography and clinical parameters, e.g., age, CFTR genotype, lung function and use of IV-antibiotics (Table 1). However, treatment with inhaled antibiotics was more common in the *Aspergillus* group (*p* < 0.05), while use of macrolide antibiotics were more common in the non-colonized group (*p* < 0.05). The association between macrolide treatment and *Aspergillus* colonization did not remain significant when potential confounders were taken into consideration, i.e., age, pancreatic insufficiency, inhaled corticosteroids, inhaled antibiotics, chronic *P. aeruginosa* infection, and ppFEV1 (odds ratio (OR) = 0.5, 95% CI 0.2–1.1) (Fig. 3). However, the association between inhaled antibiotics and *Aspergillus* colonization was still valid after adjusting for confounders, yielding an OR of 3.1 (95% CI 1.6–5.9) of becoming colonized with *A. fumigatus* for patients treated with inhaled antibiotics.

At baseline, there was a difference between the groups regarding pancreatic insufficiency, which was more commonly observed in the *Aspergillus* group (95% vs 86%, *p* < 0.05). Moreover, chronic *P. aeruginosa* colonization was more frequently observed in the non-colonized group (28% vs 42%, *p* < 0.05). However, these associations did not persist when analyses were adjusted for confounders, i.e., age, inhaled antibiotics, macrolide treatment, inhaled corticosteroids, and ppFEV1 (Fig. 3).

### Impact of *A. fumigatus* colonization on lung function and other clinical parameters

**Fig. 4:**
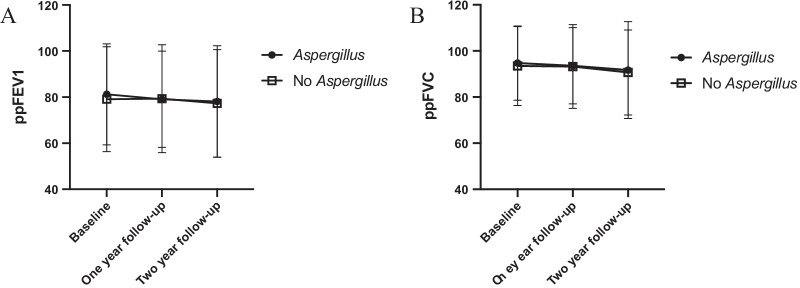
Lung function in the *Aspergillus* vs. the non-colonized groups. Lung function displayed as ppFEV1 (**A**) and ppFVC (**B**) at baseline, one year follow-up and two-year follow-up. ppFEV1 = percent predicted Forced Expiratory Volume in one second. *ppFVC* percent Forced Vital Capacity in % of predicted. Standard deviations are indicated by error bars

When comparing baseline with follow-up after two years, both the *Aspergillus* group and the non-colonized group showed a decline in ppFEV1 with a mean ∆ppFEV1 of − 3.3 (SD 11.3, *p* < 0.05) and − 2.0 (SD 10.0, *p* < 0.05), respectively. Also, both groups showed a decline in ppFVC with a mean ∆ppFVC of − 3.4 (SD 11.9, *p* < 0.05) and − 3.0 (SD 10.0, *p* < 0.05). However, there was no statistical support for a difference in lung function decline between the groups, neither for ppFEV1 nor for ppFVC (*p* = 0.22 and *p* = 0.72 respectively) (Fig. 4A, B).

The *Aspergillus*-group had more hospitalization days compared to the non-colonized group when the two follow-up years were summarized (median 0.5, IQR 0–5 vs median 0, IQR 0–2, respectively, *p* < 0.05), but the two groups did not differ in the number of IV-antibiotic treatments at one-year follow-up (median 1, IQR 0–3 vs median 1, IQR 0–3, respectively, *p* = 0.23), or at two-year follow-up (median 1, IQR 0–3 vs median 1, IQR 0–3, *p* = 0.33).

The increase in total IgE between baseline and the follow-up two years later was greater in the *Aspergillus* group compared to the non-colonized group (median 10, IQR − 4.2–24 vs median − 1, IQR – 22–15, respectively, *p* < 0.05). This difference remained significant after exclusion of *Aspergillus*-colonized patients that developed ABPA during the follow-up period (*n* = 4, *p* < 0.05). Also, the *Aspergillus* group had higher levels of eosinophil counts compared to the non-colonized group at both the one-year follow-up (median 0.2, IQR 0.1–0.4 vs median 0.2, IQR 0.1–0.3, respectively, *p* < 0.05) and at the two-year follow-up (median 0.3, IQR 0.1–0.4 vs median 0.2, IQR 0.1–0.3, *p* < 0.05), but the total increase throughout the follow-up period did not differ between the groups (median 0.0, IQR − 0.0–0.1 vs median 0.0, IQR − 0.1–0.1, *p* = 0.35).

### Treatment of asymptomatic *A. fumigatus* colonization

Of the 64 patients who became colonized with *A. fumigatus*, 22 had airway symptoms assessed to be caused by *Aspergillus* and four of the 22 individuals developed ABPA during the follow-up period. All 22 patients were treated with antifungal drugs. Comparing lung function at baseline with the follow-up two years later, there was no statistical support for a decline in either mean ppFEV1 (∆ppFEV1 =  − 0.90, *p* = 0.74) nor in mean ppFVC (∆ppFVC =  − 0.61, *p* = 0.80). From here on, the symptomatic group was excluded from further analysis.

**Fig. 5:**
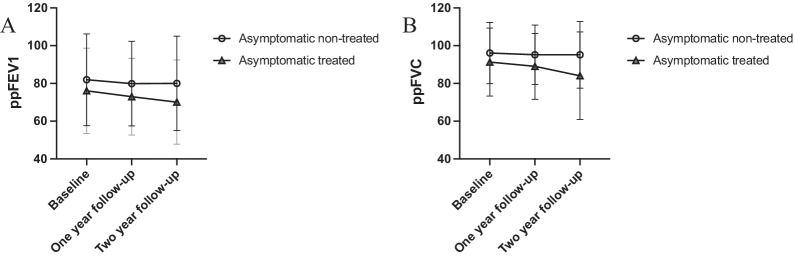
Lung function in the treated vs. the non-treated groups. Lung function displayed as ppFEV1 (**A**) and ppFVC (**B**) at baseline, one year follow-up and two-year follow-up. ppFEV1 = percent predicted Forced Expiratory Volume in one second. *ppFVC* percent predicted Forced Vital Capacity. Standard deviations are indicated by error bars

**Fig. 6:**
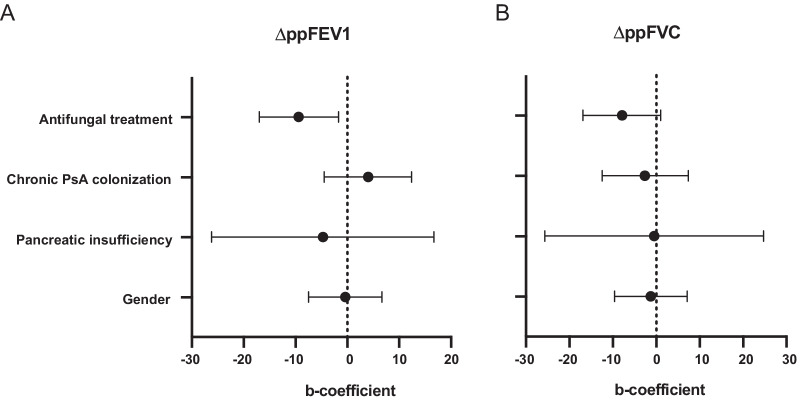
Multivariate associations between different variables and lung function decline in patients with asymptomatic *A. fumigatus* colonization. The association between different variables and (**A**) ∆ppFEV1 (difference between ppFEV1 at baseline and follow-up two years later) and (**B**) ∆ppFVC (difference between ppFVC at baseline and follow-up two years later) were estimated using multivariate linear regression. The forest plot shows unstandardized b-coefficients and 95% confidence intervals. *N* = 35. *PsA Pseudomonas* aeruginosa; *ppFEV1* percent predicted Forced Expiratory Volume in one second; *ppFVC* Forced Vital Capacity

The remaining 42 patients without clinical symptoms of infection were then divided into two groups depending on whether they received antifungal treatment or not (Fig. 2). In the treated group (*n* = 19, 45%), 17 patients were treated with Posaconazol and two with inhaled Amphotericine B. The median length of antifungal treatment was 3 months (range 1–10 months) and resulted in eradication of *A. fumigatus* in 13 patients. In 6 patients, the fungus re-appeared in airway cultures after termination of treatment. At baseline, the treated and non-treated groups were comparable both in terms of demographics and clinical parameters, except for chronic *P. aeruginosa* infection which was more commonly reported in the treated group (Table 2). When comparing the mean change in ppFEV1 between baseline and one-year follow-up, there was no significant difference between the two groups (∆ppFEV1 =  − 4.0, SD 6.7 vs − 2.5, SD 9.9, respectively, *p* = 0.59). However, when comparing ppFEV1 between baseline and the two-year follow-up the treated group decreased more in mean ppFEV1, compared to the non-treated group (∆ppFEV1 =  − 8.1, SD 9.0 vs − 1.4, SD 10.4, *p* < 0.05) (Fig. 5A). The change in ppFVC between baseline and two-year follow-up showed a similar trend, but the difference between the treated and the non-treated groups was not statistically significant (mean ∆ppFVC =  − 8.5, SD 12.1 and − 1.5, SD 11.2, *p* = 0.07) (Fig. 5B). The relationship between treatment and ∆ppFEV1 persisted when gender, pancreatic insufficiency and chronic *P. aeruginosa* infection, that are known to affect lung function [22, 23], were adjusted for in a regression model. The association between treatment and ∆ppFVC was not significant (Fig. 6).

**Table 2:** Baseline characteristics

Baseline characteristics	Asymptomatic with treatment	Asymptomatic without treatment	*p*-value
	n (%)	n (%)	
Total	19 (45)	23 (55)	
*Demographics and CF characteristics*			
Age, years (median (IQR))	19.2 (11.4–33.5)	20.2 (12.7–29.1)	0.86
Female sex	7 (37)	9 (39)	0.88
Homozygous DeltaF508	8 (42)	14 (61)	0.23
CF-related diabetes	7 (37)	6 (26)	0.45
Pancreatic insufficiency	19 (100)	21 (91)	0.49
*CF centre*			0.00
Gothenburg (*n* = 14)	0	14	
Lund (*n* = 11)	9	2	
Stockholm (*n* = 15)	10	5	
Uppsala (*n* = 2)	0	2	
*Microorganisms*			
Chronic PsA colonization	8 (42)	3 (15)	0.06
*Treatments*			
Inhaled antibiotics	9 (50)	12 (54)	0.78
Inhaled corticosteroids	2 (11)	8 (35)	0.08
Macrolides	5 (26)	3 (13)	0.43
Inhaled rhDNase	7 (37)	5 (22)	0.28
*Lung function and other clinical parameters*			
ppFEV1 (mean ± SD)	76.1 ± 22.6	82.0 ± 24.3	0.45
ppFVC (median (IQR))	100.0 (79.2–104.7)	94.7 (86.5–107.2)	0.67
Number of IV-antibiotics (median (IQR))	1 (0–3)	1 (0–3.25)	0.84
Hospitalization days (median (IQR))	0 (0–3.5)	0 (0–2.25)	0.88
Total IgE (median (IQR))^a^	18.0 (9.1–88.5)	41.0 (10.7–55.3)	0.73
Eosinophils (median (IQR))^b^	0.2 (0.1–0.4)	0.2 (0.1–0.3)	0.58

Treated vs non-treated patients with asymptomatic *A. fumigatus* colonization

*IQR* Interquartile Range; *PsA Pseudomonas aeruginosa*. *ppFEV1* percent predicted Forced Expiratory Volume in one second; *ppFVC* percent predicted Forced Vital Capacity

^a^n = 25

^b^n = 26

During the period from baseline to the two-year follow-up there were no significant differences between the treated and the non-treated groups regarding increase of eosinophil counts (median 0.1, IQR 0.0–0.5 vs median 0.0, IQR 0.0–0.1, respectively, *p* = 0.29), or in increase of total IgE (median 10, IQR – 5–24 vs median 14, IQR – 3–27, *p* = 0.82). Nor were there differences between the groups in their use of IV-antibiotics (mean 3.2, SD 2.7 vs mean 3.7, SD 3.4, *p* = 0.60), or in the number of hospital days when the two follow-up years were summarized (median 1, IQR 0–3.5 vs median 1, IQR 0–6.8, *p* = 0.77). None of the patients with asymptomatic *A. fumigatus* colonization developed ABPA during the follow-up period.

## Discussion

In this retrospective study on persistent *A. fumigatus* colonization in CF, we identified an association between inhaled antibiotics and colonization, but there were no differences in subsequent lung function decline or in the use of IV-antibiotics between colonized and non-colonized patients. Regarding asymptomatic *Aspergillus* colonization, no benefits of antifungal treatment could be demonstrated.

In accordance with the results from this study, inhaled antibiotics has previously been shown to be associated with persistent *Aspergillus spp* infection [8, 15, 22]. However, whether this association is causal or reflects disease severity is not fully understood. Previous studies have also identified pancreatic insufficiency [8], age [3], inhaled corticosteroids [8, 9, 14] and macrolides [8] as independent risk factors for colonization*.* None of these variables were associated with *A. fumigatus* colonization in this study. In fact, macrolide treatment was associated with low odds of becoming colonized with *A. fumigatus*. The use of macrolides is likely related to chronic *P. aeruginosa* colonization, both of which were more common in the *Aspergillus*-free group, since macrolide treatment is recommended for patients with chronic *P. aeruginosa* colonization [25]. In contrast, treatment with inhaled antibiotics did not appear to be related to chronic *P. aeruginosa* colonization in this study (Table 1). This was an unexpected finding, and it may be speculated that inhaled antibiotics is more commonly used for the eradication of *P. aeruginosa* than for treatment of chronic *P. aeruginosa* colonization in Sweden. Moreover, the reason why *P. aeruginosa* colonization was less common in patients with persistent *A. fumigatus* in this study is not fully understood. *P. aeruginosa* inhibits in vitro growth of *Aspergillus* via the secretion of pyoverdine and phenazines [26–28], which may provide a possible explanation. On the other hand, an epidemiological association between chronic *P. aeruginosa* and *Aspergillus* colonization has previously been reported [8]. Taken together, the diverging results reflect the difficulty in assessing the impact of different microbes in CF due to complex polymicrobial interactions in the airways and possible confounding by disease severity.

In this study, persistent *A. fumigatus* infection did not impact the patients´ lung function or number of IV-antibiotic treatments. Previous studies on *Aspergillus* in CF have reported diverging results, where some have shown that persistent *A. fumigatus* infection is associated with more frequent exacerbations [12, 13] and a lower lung function [3, 5, 12, 13], whereas other studies have not shown a relationship between *Aspergillus* colonization and lung function decline [15–17]. Although this study could not demonstrate an association between persistent *A. fumigatus* infection and lung function decline, the *Aspergillus* group had higher levels of eosinophil counts as well as a higher increase of total IgE levels compared to the non-colonized individuals. This was not only due to the fact that four patients in the *Aspergillus* group developed ABPA during the follow-up period, since the difference in total IgE remained significant even after exclusion of the ABPA patients. Although the definitions of ABPA and *Aspergillus* sensitization are difficult and don’t rely on total IgE alone, the increase in IgE levels may indicate that individuals in the colonized group have become sensitized against *A. fumigatus*. Previous work has suggested that *Aspergillus* sensitization and elevated IgE levels increase the risk of lung function decline in patients with CF [29]. In addition to differences in lab parameters, patients with persistent *A. fumigatus* also had more hospital days compared to non-colonized patients. However, it should be noted that the reasons for hospitalization are unknown and may not always be CF-related. At-home IV antibiotic treatment is common in Swedish CF care, and people with CF are not regularly admitted to hospital during pulmonary exacerbations. Consequently, the number of hospital days may not adequately reflect the burden of CF airway disease in our study.

This study is one of few that evaluates the impact of treating asymptomatic *A. fumigatus* colonization in people with CF, and the only two previous studies that have assessed antifungal treatment show conflicting results. In a randomized double-blind placebo-controlled trial, Aaron et al. studied if treatment with Itraconazole affected the clinical outcome of CF-patients with persistent *A. fumigatus* infection. No differences in lung function or frequency of exacerbations were found between the treatment and placebo groups, but the study was small and over 40% of the treated patients had inadequate serum levels of Itraconazol [18]. In contrast, Coughlan et al. conducted a small study showing that treatment with Itraconazole reduced the numbers of exacerbations and respiratory symptoms when *A. fumigatus* was eradicated in patients with CF [19]. Similar to the randomized trial by Aaron et al. [18], there were no obvious benefits of antifungal treatment in this study. Surprisingly, the results even indicated that treatment could be associated with a faster decline in lung function. The change in ppFEV1 persisted even after controlling for potentially confounding variables previously associated with worsening lung function [23, 24]. However, the results need to be interpreted with great caution given the small number of participants, and since the rate of lung function decline might be influenced by other factors than the antifungal treatment per se. Although there were no statistically significant differences in lung function or other characteristics between the treated and the non-treated group at baseline, the treated group was to a higher degree chronically colonized with *P. aeruginosa* and had a slightly lower ppFEV1. This may indicate that these individuals were already in a worse condition prior to *A. fumigatus* colonization and would in such case have a higher risk of deteriorating lung function [30]. Importantly, we lack data on the rate of lung function decline before the start of *A. fumigatus* colonization. This parameter may influence the subsequent lung function progression during the study period and potentially also the intention to treat colonized patients. There may also have been residual confounding. For example, an important and possibly confounding reason why the patients received treatment or not is the different approaches across the CF centres in this study. Treatment-prone centres may be more active in identifying *A. fumigatus* colonization, which may lead to different characteristics of colonized patients between the centres. Unfortunately, further statistical analysis to adjust for these differences was not possible due to the low number of participants.

This study has several limitations, such as the small number of *Aspergillus*-colonized patients and missing data due to the retrospective design. Importantly, the Swedish CF registry lacks information on specific antibodies against *A. fumigatus*, which is an important parameter for the characterization of ABPA and *Aspergillus* sensitization in CF [29]. Instead, we used total IgE and eosinophil counts, but specific *Aspergillus* antibodies would have allowed us to more accurately characterize and compare our cohort. We also lack data on whether or not the antifungal treatment reached adequate plasma concentrations in order to be effective. Moreover, data on chronic *Staphylococcus aureus* infection as well as other relevant pathogens were poorly reported in the registry and could not be included in the analysis. Another important limitation is the definition of symptoms related to persistent *A. fumigatus* infection. Commonly used definitions of *Aspergillus* bronchitis, including lack of response to antibacterial treatment [5], was difficult to apply to our study setting since CF centres that treat asymptomatic persistent *A. fumigatus*-infection usually initiate antifungal treatment in response to repeated growth of *A. fumigatus* in airway cultures without evaluating fungal-related symptoms or response to antibacterial treatment. Instead, we based our classification on symptoms described in the medical journal and the judgement by the responsible physician. This is a subjective assessment that may differ between physicians, and there is a risk that symptomatic patients may have been misclassified as asymptomatic and vice versa. Moreover, the study has a relatively short follow up-time of only two years, and we have not adjusted for the duration of *Aspergillus* colonization or whether or not eradication of *A. fumigatus* was successful in the treated group. In a previous study on *Aspergillus* colonization in CF, the duration of colonization did not affect the degree of lung function decline after adjusting for confounders [15]. Even so, it would be valuable to study if treatment of asymptomatic *A. fumigatus* colonization makes a difference in a long-term perspective with regards to lung function and ABPA. Finally, there is no uniform definition of persistent *A. fumigatus* infection in CF research or in the Swedish CF registry. We used a definition commonly used in other studies [3, 8, 12], and to overcome the uncertainty of relying on registry data alone, *A. fumigatus* colonization status, fungal treatment, and airway symptoms were validated in the patients’ medical records. Consequently, the *Aspergillus* group in this study was well defined and the risk of misclassification is low. However, there is a possibility that some *Aspergillus*-colonized patients may have been misclassified into the non-colonized group due to potentially missing data on *Aspergillus* findings in the registry. If so, this misclassification would likely bias the estimates towards the null.

## Conclusions

This study shows that treatment with inhaled antibiotics is associated with increased odds of becoming colonized with *A. fumigatus* in people with CF. We could not demonstrate any clinical benefit of treating asymptomatic *A. fumigatus* colonization, but the study was small and lacked statistical power. Future studies on treatment of asymptomatic *A. fumigatus* colonization would benefit from a larger cohort and better controlling for built-in confounders through matching or randomization.

## Data Availability

The datasets used and/or analyzed during the current study are available from the corresponding author on reasonable request.

## Abbreviations

ABPA: Allergic bronchopulmonary aspergillosis
CF: Cystic fibrosis
CI: Confidence interval
CFTR: Cystic fibrosis transmembrane conductance regulator
ppFEV1: Percent predicted Forced expiratory volume in one second
∆ppFEV1: The change in lung function calculated as the difference in ppFEV1 between baseline and follow-up and expressed as percentage points.
ppFVC: Percent predicted Forced vital capacity
∆ppFVC: The change in lung function calculated as the difference ppFVC between baseline and follow-up and expressed as percentage points.
IQR: Interquartile range
OR: Odds ratio
SD: Standard deviation
VIF: Variance inflation factor
